# Provision of Stroke Care Services by Community Disadvantage Status in the US, 2009-2022

**DOI:** 10.1001/jamanetworkopen.2024.21010

**Published:** 2024-07-25

**Authors:** Renee Y. Hsia, Nandita Sarkar, Yu-Chu Shen

**Affiliations:** 1 Department of Emergency Medicine, University of California, San Francisco; 2 Philip R. Lee Institute for Health Policy Studies, University of California, San Francisco; 3 National Bureau of Economic Research, Cambridge, Massachusetts; 4 Department of Defense Management, Naval Postgraduate School, Monterey, California

## Abstract

**Importance:**

Stroke center certification is granted to facilities that demonstrate distinct capabilities for treating patients with stroke. A thorough understanding of structural discrimination in the provision of stroke centers is critical for identifying and implementing effective interventions to improve health inequities for socioeconomically disadvantaged populations.

**Objective:**

To determine whether (1) hospitals in socioeconomically disadvantaged communities (defined using the Area Deprivation Index) are less likely to adopt any stroke certification and (2) adoption rates differ between entry-level (acute stroke–ready hospitals) and higher-level certifications (primary, thrombectomy capable, and comprehensive) by community disadvantage status.

**Design, Setting, and Participants:**

This cohort study used newly collected stroke center data merged with data from the American Hospital Association, Healthcare Cost Report Information datasets, and the US Census. All general acute hospitals in the continental US between January 1, 2009, and December 31, 2022, were included. Data analysis was conducted from July 2023 to May 2024.

**Main Outcomes and Measures:**

The primary outcome was the likelihood of hospitals adopting stroke care certification. Cox proportional hazard and competing risk models were used to estimate the likelihood of a hospital becoming stroke certified based on the socioeconomic disadvantage status of the community.

**Results:**

Among the 5055 hospitals studied from 2009 to 2022, 2415 (47.8%) never achieved stroke certification, 602 (11.9%) were certified as acute stroke–ready hospitals, and 2038 (40.3%) were certified as primary stroke centers or higher. When compared with mixed-advantage communities, adoption of any stroke certification was most likely to occur near the most advantaged communities (hazard ratio [HR], 1.24; 95% CI, 1.07-1.44) and least likely near the most disadvantaged communities (HR, 0.43; 95% CI, 0.34-0.55). Adoption of acute stroke–ready certification was most likely in mixed-advantage communities, while adoption of higher-level certification was more likely in the most advantaged communities (HR,1.41; 95% CI, 1.22-1.62) and less likely for the most disadvantaged communities (HR, 0.31; 95% CI, 0.21-0.45). After adjusting for population size and hospital capacity, compared with mixed-advantage communities, stroke certification adoption hazard was still 20% lower for relatively disadvantaged communities (adjusted HR, 0.80; 95% CI, 0.73-0.87) and 42% lower for the most disadvantaged communities (adjusted HR, 0.58; 95% CI, 0.45-0.74).

**Conclusions and Relevance:**

In this cohort study examining hospital adoption of stroke services, when compared with mixed-advantage communities, hospitals located in the most disadvantaged communities had a 42% lower hazard of adopting any stroke certification and relatively disadvantaged communities had a 20% lower hazard of adopting any stroke certification. These findings suggest that there is a need to support hospitals in disadvantaged communities to obtain stroke certification as a way to reduce stroke disparities.

## Key Points

**Question:**

What is the association of community socioeconomic disadvantage with the likelihood of nearby hospitals adopting stroke center certification?

**Findings:**

In this cohort study of 5055 US hospitals from 2009 to 2022, hospitals located near socioeconomically disadvantaged communities were 20% to 42% less likely to become stroke certified compared with hospitals near mixed-advantage communities.

**Meaning:**

In this cohort study, hospitals in communities with the greatest level of socioeconomic disadvantage had the lowest likelihood of becoming stroke certified while hospitals in the most advantaged communities had the highest likelihood; these findings illuminate persistent structural disparities in the geographic distribution of certified stroke centers.

## Teaser

This cohort study of US hospitals analyzes the likelihood of hospitals adopting stroke certification status based on the socioeconomic disadvantage status of the surrounding community.

## Introduction

The National Institute of Minority Health and Health Disparities (NIMHD) has identified that access to health care services and technology may be a specific—and intervenable—mechanism by which disadvantaged communities benefit differently from their advantaged counterparts.^1^ The NIMHD has called for rigorous evaluation of structural determinants and disadvantage,^2^ such as the *built environment*—or the human-made physical infrastructure that supports human activity—which has been associated with various health outcomes.^3,4,5^ In particular, disparities in access to health care and treatment stem from the intricate interplay between the availability and geographic distribution of these services.^6,7^

Stroke centers are acute care hospitals that have the ability to provide specialized stroke services; these centers are sometimes certified by national certification organizations but also by state bodies. The stroke care system is an opportune model for evaluating structural inequities in access to stroke care and the corresponding association with the health of individuals and communities. Stroke center certification, a critical component of stroke systems, was introduced by The Joint Commission in 2004 to improve the quality and coordination of acute stroke care. Since then, hospitals have been able to obtain stroke certification at varying levels through several different national certifying organizations, ranging from acute stroke–ready hospital (ASRH) to primary stroke center (PSC), thrombectomy-capable stroke center (TSC), and comprehensive stroke center (CSC). Receiving care at these certified stroke centers has been associated with improved outcomes for patients with acute stroke.^8,9,10,11^

Few studies have examined how structural racism and discrimination—“the macro-level conditions that limit opportunities, resources, power, and well-being of individuals and populations based on race/ethnicity and other statuses”^12^—operate in the provision of health care services, and specifically stroke care. This is an area that demands greater attention, as it is well established that structural racism and discrimination are associated with disparities in stroke incidence and outcomes.^13,14,15,16^ Analyses of access to health care services, such as emergency departments and cardiac care, have previously established that hospitals serving more affluent or commercially insured patients have a higher likelihood of adopting these services, and that poorer areas have a higher likelihood of losing these services. The few structural studies^17,18,19^ on geographic access to stroke care that exist have shown—contrary to our hypothesis—that Black and Hispanic patients have better access to stroke centers than White patients. While important, these studies are cross-sectional and use 1-dimensional measures of demographic factors to estimate geographic access to stroke care without controlling for population density. Our initial work in this area found that, after controlling for population size, hospitals in low-income communities and Black, racially segregated communities were less likely to obtain stroke certification.^20,21^ Another limitation of existing studies is the reliance on a single dimension to characterize communities’ disadvantage status (eg, income or race and ethnicity), which may mask the complicated realities of the intersection of socioeconomic factors that affect people’s lives.

Using data from all hospitals across the US and newly collected stroke certification data from not only national certifying organizations, but also state certifying departments from 2009 to 2022, we address the following research questions: (1) whether hospitals in disadvantaged communities (as defined by the Area Deprivation Index [ADI]) are less likely to adopt any stroke certification and (2) whether adoption rates of lower and higher level stroke certification differ between hospitals in disadvantaged and nondisadvantaged communities. This work builds upon our prior work^21^ by stratifying outcomes by stroke certification level rather than a cruder binary indicator for presence of stroke center. Using the ADI to capture the multidimensional socioeconomic conditions of communities improves upon previous analyses that used only a single measure of historical disadvantage (eg, race or income) and is better suited for identifying communities in need of targeted intervention to reduce disparities. This study also utilizes a more granular zip code rather than larger hospital service areas (HSAs) in previous work. There are about 3000 HSAs and 40 000 zip codes, so the disadvantaged communities defined in this study are much more localized. Without a clear understanding of where to deploy resources, and the degree to which disparities could be changed, effective policy interventions to dismantle structural discrimination in stroke care cannot be developed, and narrowing the gap in health outcomes for racial and ethnic minorities and patients with low socioeconomic status will remain out of reach.^22^

## Methods

### Data

This cohort study was approved through the National Bureau of Economic Research and followed the Strengthening the Reporting of Observational Studies in Epidemiology (STROBE) reporting guideline. The study universe included all general acute nonfederal hospitals in the continental US. From January 1, 2009, to December 31, 2022, we obtained comprehensive data on stroke certification from 4 Centers for Medicare & Medicaid Services (CMS)–approved national certifying organizations and from 14 states that were determined to have their own state-run self-certification or independent designation process for stroke centers, meaning the state offers an option for hospital stroke center certification without reliance on a national certification. Certification data for hospitals in these 14 states were collected from individual states; further details on data collection methods and state-specific certification levels are included in the eMethods and eTable 1 in Supplement 1, and elsewhere.^23^ Data for all stroke centers, regardless of certification process, included the level of certification for each hospital and the date the certification became effective. These data were merged with American Hospital Association annual surveys and the Healthcare Cost Report Information dataset to obtain additional facility data, such as geographic location (address and longitude and latitude coordinates), bed size, occupancy rate, hospital ownership, whether the hospital was part of a system, and teaching status. We further supplemented the facility data with community level data from the US Census (to identify longitude and latitude of a neighborhood’s population center) and the Neighborhood Atlas (to identify the socioeconomic status of the neighborhoods surrounding each hospital).^24,25^

### Identifying Hospital Stroke Care Capacity

We identified different levels of stroke care based on certification data from 4 CMS-approved accrediting organizations: The Joint Commission, Det Norske Veritas, Accreditation Commission for Health Care (formerly the Healthcare Facilities Certification Program), and Center for Improvement in Healthcare Quality, as well as state departments of health. Certification levels and the corresponding terminology have expanded over time, and nomenclature varies slightly across national certifying organizations and states. Following extensive surveys and consultation with stakeholders^26^ and protocols employed by national certifying organizations, we grouped stroke certification levels into the following categories (from least to most advanced): ASRH, PSC, TSC, and CSC.

### Identifying Hospital Neighboring Community Socioeconomic Status

We captured the socioeconomic conditions of neighborhoods surrounding each hospital based on the percentage of residents living with a high or low ADI—a validated marker of neighborhood-level socioeconomic disadvantage that combines 17 measures of employment, income, housing, and education from the American Community Survey—to create a summary score for each census block group (CBG) in the US.^24,25,27,28,29,30,31^ Because ADI is defined at the CBG level, we defined surrounding neighborhoods as all CBGs within a 15-minute driving time of a given hospital. We believe this small geographic coverage best captures the surrounding local socioeconomic conditions.^32,33,34^

We classified a CBG as a high ADI neighborhood (ie, highly disadvantaged neighborhood) if its nationally ranked ADI value was at the 80th percentile or above and as a low ADI neighborhood (ie, highly advantaged) if the nationally ranked ADI value was at the 20th percentile or below. For each hospital, we first identified all CBGs that were within a 15-minute driving time under normal traffic conditions.^35,36^ We then categorized hospitals into 5 groups based on the share of the surrounding population in low or high-ADI neighborhoods. Specifically, hospitals were said to be located in the most advantaged communities if more than 50% of the surrounding population was living in low ADI neighborhoods; likewise, the most disadvantaged communities were defined as those with more than 50% of the population in high ADI neighborhoods. Hospitals located in relatively advantaged communities had 25% to 50% of the population in low ADIs, and relatively disadvantaged communities had 25% to 50% of the population in high ADIs. The rest of the hospitals (those where surrounding neighborhoods had less than 25% of the population in high and low ADIs) were defined as located in mixed-advantage communities. To track the surrounding neighborhoods consistently over time, these community ADI categories were made time-invariant based on ADI values from 2015 (the earliest year available). Our past experience working with these types of community-level measures has demonstrated that they are highly correlated over time.^37^

### Statistical Analysis

Following prior literature,^20,21,38^ we estimated a hospital’s adoption of stroke certification using a survival analysis framework. In model 1, we estimated a hospital’s overall adoption of any stroke certification (regardless of level) using a Cox proportional hazard model.^38,39,40^ In our context, the hazard was defined as the instantaneous probability that hospital *j* received stroke care certification starting at quarter *t*, conditional on having no such capacity up until time *t*. The dependent variable took on a value of 1 during the first quarter that hospital *j* attained any level of stroke care. Hospitals that did not adopt any stroke care certification at the end of the study period (or the last quarter they operated, whichever came first) were censored. The key independent variables were the set of indicators capturing the disadvantage status of each hospital’s surrounding neighborhoods as defined above. In model 1A, we included the disadvantage status dummies and an indicator for rural locations to compare differential hazard rates purely from a location perspective. In model 1B, we included additional controls that accounted for HSA population size and hospital size. Controlling for population size is important in this context because competition for hospital access would be greater for an area with a potential service population of 1 million rather than 1000. In essence, model 1B compares the differences in per-capita stroke care adoption rates across hospitals.

In model 2, we explored whether adoption rates for lower and higher levels of stroke care differed for hospitals located in disadvantaged communities compared with those in nondisadvantaged communities. Specifically, we expanded model 1 to separately estimate adoption of ASRH vs higher level stroke hospitals (PSC or above) in a competing-risks framework.^41^ This is because ASRHs, in general, provide the most basic stroke services including rapid stroke assessment, stabilization, and administration of tissue plasminogen activator before transferring patients to higher level centers (PSC or above) for more advanced care.^42^ The covariates for these competing risk models are the same as models 1A and 1B, but model 2 treats the alternative certification level as a competing event rather than standard censoring.

Data were analyzed using Stata version 18 (StataCorp). Results were deemed significant at *P* < .05 using 2-sided tests. Data analysis occurred from July 2023 to May 2024.

## Results

A total of 5055 unique US hospitals that operated between 2009 to 2022 were included in the study, of which 2640 (52.2%) had attained some level of stroke certification. In 2009, of the 5055 hospitals, 316 (6.3%) were located in the most advantaged communities, 529 (10.5%) in relatively advantaged, 1954 (38.7%) in mixed-advantage, 1841 (36.4%) in relatively disadvantaged, and 359 (7.1%) in the most disadvantaged. A total of 912 hospitals (18.0%) in our analysis were not present in all quarters. Hospital ownership varied significantly; of all hospitals, 2864 (56.7%) were not-for-profit, 835 (16.5%) were for-profit, and 1105 (21.9%) were government owned. A total of 1294 hospitals (25.6%) were critical access hospitals and 304 (6.0%) were teaching hospitals. When looking at stroke center certification, 2415 hospitals (47.8%) never achieved stroke certification during the study period, while 602 (11.9%), attained ASRH status, and 2038 (40.3%) obtained PSC status or higher (Table). A total of 79 hospitals (1.6%) attained ASRH status prior to attaining PSC status (analyzed as part of the PSC category). When looking at hospitals that attained a higher level of certification (PSC or above), 185 (9.1%) were located in communities categorized as most advantaged and 344 (16.9%) were located in relatively advantaged communities, while only 26 (1.3%) were located in most disadvantaged communities. In contrast, among hospitals that never had any stroke certification during the 14-year period, only 114 (4.7%) were located in the most advantaged communities and 289 (12.0%) were in the most disadvantaged communities.

**Table.** Hospital Characteristics During Study Period

Hospital characteristic	Hospitals by certification, No. (%)			
	All hospitals (N = 5055)	Never had stroke certification during study period (n = 2415)	Attained acute stroke–ready hospital as highest level during study period (n = 602)	Attained primary stroke center or higher during study period (n = 2038)
Socioeconomic status of hospital neighborhood				
Most advantaged	316 (6.3)	114 (4.7)	17 (2.8)	185 (9.1)
Relatively advantaged	529 (10.5)	147 (6.1)	38 (6.3)	344 (16.9)
Mixed-advantage community	1954 (38.7)	854 (35.4)	263 (43.7)	837 (41.1)
Relatively disadvantaged	1841 (36.4)	957 (39.6)	238 (39.5)	646 (31.7)
Most disadvantaged	359 (7.1)	289 (12.0)	44 (7.3)	26 (1.3)
Location				
Stroke belt states	869 (17.2)	417 (17.3)	148 (24.6)	304 (14.9)
Rural hospitals	2400 (47.5)	1679 (69.5)	401 (66.6)	320 (15.7)
Ownership				
Not-for-profit	2864 (56.7)	1119 (46.3)	359 (59.6)	1386 (68.0)
For-profit	835 (16.5)	439 (18.2)	44 (7.3)	352 (17.3)
Government	1105 (21.9)	712 (29.5)	159 (26.4)	234 (11.5)
Critical access hospital	1294 (25.6)	1008 (41.7)	255 (42.4)	31 (1.5)
No. of hospital beds				
<100	2382 (47.1)	1700 (70.4)	424 (70.4)	258 (12.7)
100-399	1748 (34.6)	326 (13.5)	110 (18.)	1312 (64.4)
≥400	374 (7.4)	13 (0.5)	3 (0.5)	358 (17.)
Teaching hospital	304 (6.0)	33 (1.)	7 (1.2%)	264 (13.0)
Has cardiac capacity^a^	2012 (39.8)	334 (13.8)	120 (19.9)	1558 (76.4)
Member of a hospital system	2709 (53.6)	1073 (44.4)	271 (45.0)	1365 (67.0)
Total inpatient discharges, median (IQR)	3600.87 (948.60-10 496.76)	1102.02 (434.12-2542.97)	1703.17 (622.71-3843.53)	11 417.78 (6634.68-18 600.46)
Case mix index, mean (SD)	1.37 (0.28)	1.21 (0.29)	1.20 (0.16)	1.48 (0.23)
Occupancy rate, mean (SD)	0.50 (0.21)	0.39 (0.19)	0.44 (0.18)	0.64 (0.15)

^a^ Either a cardiac catheterization laboratory or coronary artery bypass graft.

There were several other notable differences between hospitals that attained PSC or higher certification and those that never achieved stroke center certification. First, among hospitals that never attained stroke certification, 1679 (69.5%) were rural hospitals. There were 401 rural hospitals that achieved ASRH status (66.6%) and 320 rural hospitals that achieved PSC and higher (15.7% of all hospitals that achieved PSC and higher). Of the 602 hospitals that achieved ASRH status, only 17 (2.8%) and 38 (6.3%) were located in the most and relatively advantaged communities, while 238 (39.5%) and 44 (7.3%) were located in relatively and most disadvantaged communities. Furthermore, government ownership was much higher for hospitals that never offered specialized stroke services (712 hospitals [29.5%]) compared with hospitals that attained PSC status or above (234 hospitals [11.5%]).

**Figure 1.**
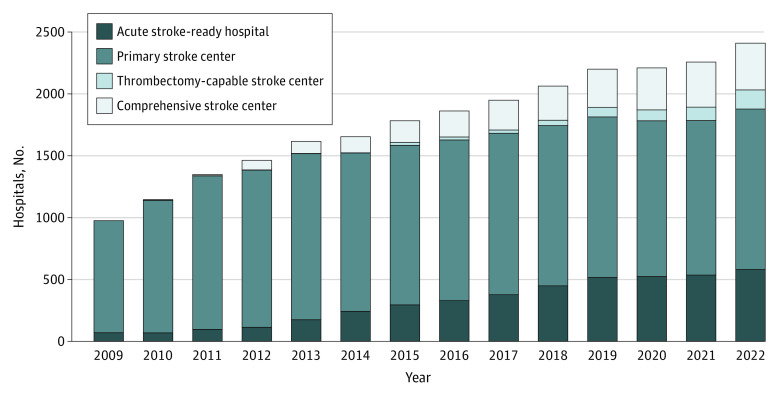
Stroke-Certified Hospitals by Type, 2009-2022

Figure 1 shows the growth of stroke certification by level between 2009 and 2022. By the end of the study period, PSCs comprised the largest proportion of stroke centers (1337 of 2449 stroke centers [54.6%], followed by ASRHs (602 of 2449 stroke centers [24.6%]), CSCs (378 of 2449 stroke centers [15.4%]), and TSCs (154 of 2449 stroke centers [6.3%]).

**Figure 2.**
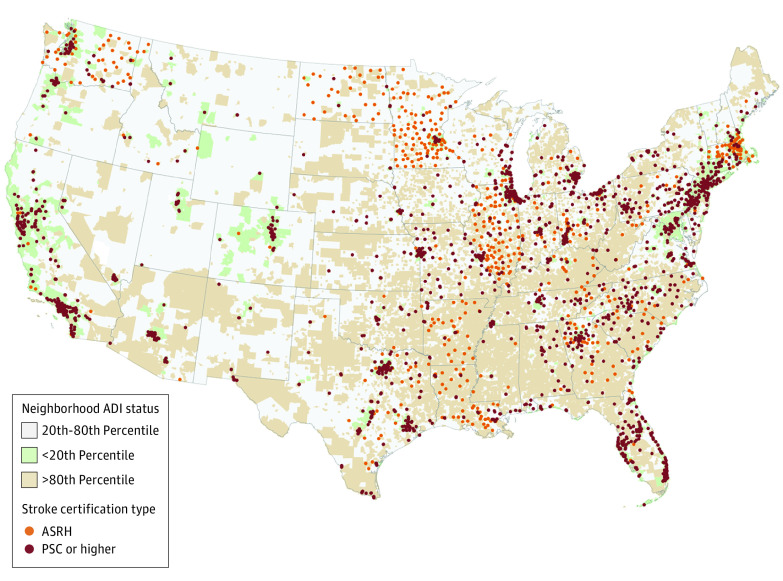
Hospital Location by Stroke Certification Type and Neighborhood Area Deprivation Index (ADI) in 2022 ASRH indicates acute stroke–ready hospital; PSC, primary stroke center.

Figure 2 shows the location of these hospitals across the US at the end of 2022. Stroke hospitals that attained PSC or above status appeared to cluster around neighborhoods classified as the most advantaged (lowest 20th percentile of ADI). Stroke hospitals that achieved ASRH status were more spread out around disadvantaged neighborhoods (ADI between the 20th and 80th percentile or above the 80th percentile).

**Figure 3.**
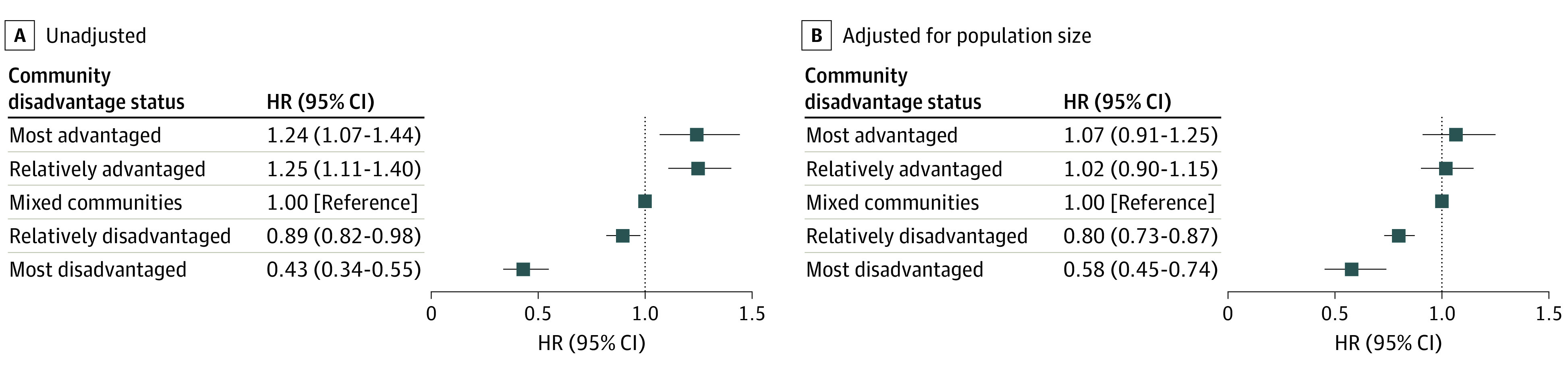
Adoption of Any Level of Stroke Certification by Community Disadvantage Status The figure shows unadjusted hazard ratios (HRs; A) and HRs adjusted for population size and hospital capacity (B). Error bars represent 95% CIs.

Figure 3A (complete regression results in eTable 2 in Supplement 1) shows that before adjusting for population size and hospital capacity, the likelihood of adopting any level of stroke certification was highest for hospitals located in the most advantaged communities (hazard ratio [HR], 1.24; 95% CI, 1.07-1.44) and relatively advantaged communities (HR, 1.25; 95% CI, 1.11-1.40). In other words, hospitals in the most advantaged communities were 1.24 times more likely than hospitals in mixed-advantage communities to adopt stroke certification at any level. On the other hand, being located in a relatively or most disadvantaged community reduced the hazard of adopting stroke certification by 11% (HR, 0.89; 95% CI, 0.82-0.98) and 57% (HR, 0.43; 95% CI, 0.34-0.55), respectively, compared with hospitals in mixed-advantage communities.

After adjusting for population and hospital capacity, however, the hazard of adoption was similar between both types of advantaged communities and the mixed-advantage (reference) communities (Figure 3B; full results in eTable 2 in Supplement 1). In other words, residents in these 3 types of communities had similar access to stroke hospitals. However, we continued to observe the sliding scale of lower adoption hazard in disadvantaged communities; compared with hospitals in mixed-advantage communities, the hazard of adopting stroke certification of any kind was 20% lower for hospitals in relatively disadvantaged communities (adjusted HR [aHR], 0.80; 95% CI, 0.73-0.87) and 42% lower for hospitals in the most disadvantaged communities (aHR, 0.58; 95% CI, 0.45-0.74).

**Figure 4.**
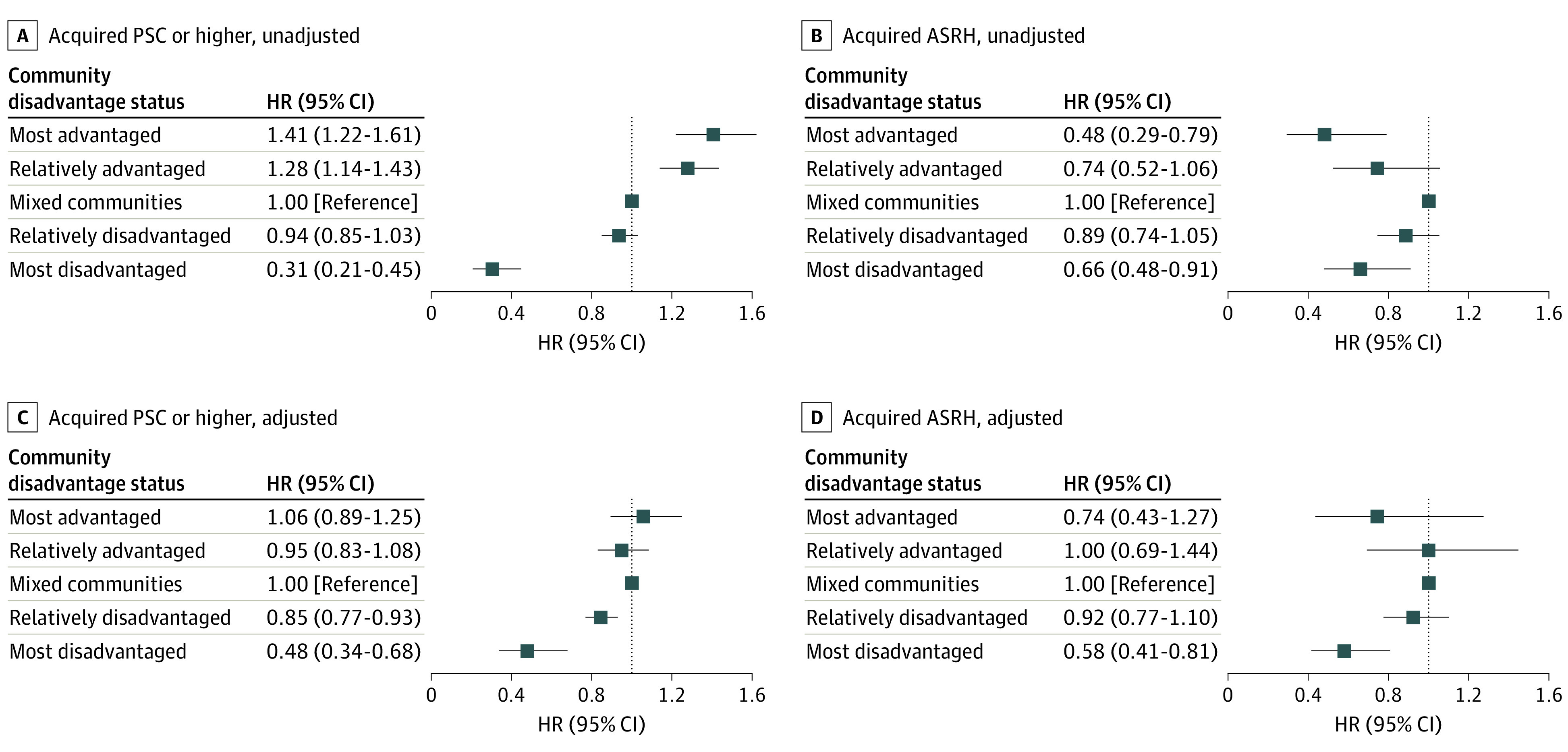
Competing Risk Model Results: Adoption of Primary Stroke Center (PSC) and Above vs Acute Stroke–Ready Hospital (ASRH) Certification Levels The figure shows unadjusted hazard ratios (HRs; A, B) and HRs adjusted for population size and hospital capacity (C, D). Error bars represent 95% CIs. PSC or higher includes PSC, Thrombectomy-Capable Stroke Center, and Comprehensive Stroke Center.

Figure 4, panels A and B (complete regression results in eTable 2 in Supplement 1) show that the unadjusted hazard of adopting PSC or higher certification exhibited a downward gradient from most advantaged (HR, 1.41; 95% CI, 1.22-1.61) to most disadvantaged (HR, 0.31; 95% CI, 0.21-0.45); whereas the hazard of adopting ASRH exhibited a C-shape that was lower in the most advantaged communities (HR, 0.48; 95% CI, 0.29-0.79), and also lower in the most disadvantaged communities (HR, 0.66; 95% CI, 0.48-0.91) compared with mixed-advantage communities. After adjusting for population size and hospital capacity, Figure 4, panels C and D show that hospitals in the most disadvantaged communities had a much lower hazard of adopting either form of stroke certification (aHR for PSC or higher, 0.48; 95% CI, 0.34-0.68; aHR for ASRH, 0.58; 95% CI, 0.41-0.81).

In our main models, we defined a hospital’s local community as the neighborhoods within a 15-minute driving time from the hospital. While such a definition can capture the socioeconomic conditions of the surrounding population, it may be too narrow to capture the relevant population that the hospital serves. In our sensitivity analysis, we broadened the geographic coverage to include all residents within a hospital’s HSA and our results were robust to this broader geographic coverage (eTable 3 in Supplement 1).

## Discussion

In our 14-year longitudinal cohort study of hospital stroke service adoption across the US, we found that even after adjusting for population size and hospital capacity, compared with hospitals in mixed-advantage communities, hospitals located in the most disadvantaged communities had a 42% lower likelihood of adopting stroke certification and relatively disadvantaged communities had a 20% lower likelihood of adopting stroke certification. Geographically, hospitals in the most and relatively advantaged communities were less likely to adopt ASRH certification; however, in our fully adjusted model, these hospitals had adoption rates similar to those in mixed-advantage communities.

The differential adoption rate of stroke care by hospitals in disadvantaged communities compared with other more advantaged communities reveals disparities in potential access to specialized care. Much of the literature on stroke disparities has focused on proximal individual-based factors, such as interpersonal discrimination, rather than the broader health impacts of structural discrimination; this may be due to perceptions that individual-level risk factors are more amenable to intervention.^43,44,45,46,47^ Our findings provide evidence that the pathway underlying well-documented differences in access, treatment, and outcomes for racial and ethnic minorities, socioeconomically disadvantaged, and rural patients who experience stroke are, in fact, upstream at the community level. In other words, disadvantaged communities have been left behind in gaining access to stroke care. Our results provide sobering evidence that socioeconomic inequality, as measured by the multidimensional metric of ADI, is associated with these structural disparities in geographic access.

What are the implications of these findings? Rather than focusing on individual-level behaviors on the part of patients or clinicians, our findings can inform the adoption of broad-based societal and policy interventions at the local, state, and federal levels to promote equal opportunity and access to health-enhancing resources among communities in need. Specifically, rather than focusing on a single measure of historical disadvantage (eg, race or ethnicity), it may yield more benefit to use a multidimensional socioeconomic metric to identify communities in need of targeted intervention to reduce disparities. While there are ongoing efforts to document the benefits of access to stroke centers at the population level in terms of both process (eg, receipt of thrombolytics or mechanical embolectomy) and outcomes (eg, mortality) measures, any potential benefits become moot if there are no stroke centers accessible to patients in disadvantaged communities.

With this in mind, there are 2 important policy implications. First would be incentivizing hospitals to form regional networks to better coordinate the transfer of patients with stroke so these patients can receive the necessary resources (eg, mechanical thrombectomy or more intensive monitoring) as needed. This is not easy when hospital systems are in competition with each other, as current literature has described, and there are clear differences in transfer based on insurance status.^48^ While hospital affiliation may be one of the strongest factors associated with transfer destination for patients with stroke, it is possible that increased protocolization with clear transfer agreements could reduce disparities. Second, there may also be a need to incentivize hospitals in disadvantaged communities to pursue stroke certification, so that they can better measure and provide the standard of care required by certification guidelines. At the state level, for example, legislation and policies involving alternate options for stroke care beyond the hospital setting (such as deployment of telestroke or mobile stroke units) might incorporate provisions that address the lack of access to stroke hospitals in disadvantaged communities. At the federal level, stroke certification is currently determined by national certifying organizations (eg, The Joint Commission or Det Norske Veritas) that have been approved by CMS, and certification hinges on individual hospital capabilities rather than specific measures that account for community needs or benefits. Improving access to stroke care for health disparity populations may require reforming existing legal and regulatory frameworks that govern the licensing, credentialing, reimbursement, and liability of stroke centers.^49,50,51^ Specifically, this could include revising the definitional requirements of stroke centers to incorporate more targeted, community-based equity measures.

While legislative and governmental interventions may seem difficult to achieve, it may be precisely these issues of governance and legislation that create the structural challenges and impose downstream barriers that impact the ability of all people to have equal opportunity for health.^52^ Just as clinical interventions for stroke have largely been funded by the public sector, increasing public funding for these structural issues of access can provide critical knowledge to guide appropriate implementation of how specialized stroke centers can achieve the best outcomes for the population. Ultimately, identifying structural mechanisms that underlie disparities in acute care systems such as stroke care has implications for other resource-intensive and technologically driven processes such as trauma care, cardiac care, and care for other time-sensitive illnesses.^52,53^

### Limitations

This study has several important limitations. First, while we were able to collect stroke certification data from national certifying organizations and most states, there were measurement errors due to reporting errors, missing data, and diminished data accuracy in earlier periods. However, we do not expect these errors to systematically differ along the community disadvantage gradient and therefore any biases in our estimation should be minimal. Second, our analysis did not account for access to telestroke services. However, a 2019 study^54^ found that just 36% of non–stroke centers had these services compared with 63% of ASRHs and 58% of PSCs, suggesting that much of telestroke expansion has occurred in areas that already have geographic access to a stroke center. Third, there were measurement errors in the disadvantage status given that inclusion of CBGs for a given hospital depends on (1) the geographic coordinates of the CBG population center and (2) driving time queries that assume normal traffic. These 2 sources of error create attenuation bias, which made our estimated HRs conservative, or smaller, than they should be. Fourth, there were 79 hospitals (1.6% of sample) that attained ASRH status prior to attaining PSC status. In the competing risk model, we categorized these hospitals in the PSC category, but our results remained robust when we categorized them in the ASRH category. Fifth, we had unbalanced panel data and 18.0% of the hospitals in our analysis were not present in all quarters. Factors that influenced a hospital’s decision to seek stroke certification might also influence their entry and exit decisions. In a sensitivity analysis, we excluded these late entrants and early exit hospitals and based our analysis on the balanced sample, and our results remained robust. Sixth, it is important to recognize that we focused on access barriers of the built environment but did not address other nongeographical barriers to care. It is likely that other barriers, such as social or structural discrimination, may deter some patients from receiving care at a stroke-certified hospital even when such a facility is in close proximity.

## Conclusions

Our cohort study of stroke certification in US hospitals from 2009 to 2022 found that hospitals in communities with the greatest level of disadvantage had the lowest likelihood of adopting specialized stroke care services while those in the most advantaged communities had the highest likelihood. Given increasing evidence showing that stroke-certified hospitals are associated with improved care, incentivizing and providing support for hospitals in disadvantaged communities to obtain stroke certification may serve as an important strategy for reducing stroke disparities.

## Notes

Supplement 1. **eMethods.** Stroke Center Data Collection Process **eTable 1.** Comparable Stroke Center Certification and Designation Levels Across National Certifying Organizations and States **eTable 2.** Complete Regression Results of the Models Presented in Figures 3 and 4 **eTable 3.** Hazard Ratios for Stroke Certification Adoption When Geographic Coverage Is Expanded to Include All Residents Within a Hospital Service Area

Supplement 2.
Data Sharing Statement
