# Error in Results

**DOI:** 10.1001/jamanetworkopen.2024.42914

**Published:** 2024-10-09

In the Original Investigation titled “Provision of Stroke Care Services by Community Disadvantage Status in the US, 2009-2022,”^1^ published July 25, 2024, there was an error in the Results section in the paragraph discussing Figure 1; the reported number of acute stroke–ready hospitals, 602 of 5055 stroke centers (24.9%), was incorrect. This should be 602 of 2449 stroke centers (24.6%). This article has been corrected.^1^
