# Supplementary material for: Provision of Stroke Care Services by Community Disadvantage Status in the US, 2009-2022
Source: JAMA Netw Open. 2024 Jul 25;7(7):e2421010. doi: 10.1001/jamanetworkopen.2024.21010 (PMC11273237; doi:10.1001/jamanetworkopen.2024.21010)
Supplement: Supplement 1. — eMethods. Stroke Center Data Collection Process eTable 1. Comparable Stroke Center Certification and Designation Levels Across National Certifying Organizations and States eTable 2. Complete Regression Results of the Models Presented in Figures 3 and 4 eTable 3. Hazard Ratios for Stroke Certification Adoption When Geographic Coverage Is Expanded to Include All Residents Within a Hospital Service Area [file jamanetwopen-e2421010-s001.pdf]

Supplemental Online Content

Hsia RY, Sarkar N, Shen Y-C. Provision of stroke care services by community disadvantage status. *JAMA Netw. Open*. 2024;7(7):e2421010. doi:10.1001/jamanetworkopen.2024.21010

eMethods. Stroke Center Data Collection Process
eTable 1. Comparable Stroke Center Certification and Designation Levels Across National Certifying Organizations and States
eTable 2. Complete Regression Results of the Models Presented in Figures 3 and 4
eTable 3. Hazard Ratios for Stroke Certification Adoption When Geographic Coverage Is Expanded to Include All Residents Within a Hospital Service Area

This supplemental material has been provided by the authors to give readers additional information about their work.

© 2024 Hsia RY et al. *JAMA Network Open.*

eMethods. Stroke Center Data Collection Process

We undertook a multi-step process to ensure we accurately captured the U.S. stroke center landscape. To identify stroke centers certified by one of the four Centers for Medicare and Medicaid Services-approved certifying organizations, we either a) identified a publicly available list of certified stroke centers on the organization's webpage (i.e., The Joint Commission), or b) contacted a representative at the organization to acquire a list of current and historical stroke centers (i.e., Det Norske Veritas, Accreditation Commission for Health Care, and Center for Improvement in Healthcare Quality). We also identified 14 states that offered an independent state process for stroke center certification (i.e., stroke centers could be self-certified/independently designated by the state without a national certification). To identify these self-certified/independently designated stroke centers, we took the following steps:

1. From September to October 2022, we conducted internet searches of public-facing webpages and administered a survey instrument to PIs at the 25 National Institutes of Health StrokeNet Regional Coordinating Centers around the country to obtain preliminary information on state certification or designation offerings.
2. We proceeded to categorize states into two groups: states with a self-certification or independent designation process and those without.
3. From October 2022 through April 2023 we verified the preliminary categorizations of state processes from Step 3 using peer-reviewed literature, primary source documents from states (e.g., state policy documents or legislation), and/or direct contact with state officials.
4. For states preliminarily determined to have a self-certification or independent designation process, we confirmed with a state official whether there was an independent state process and requested a current and historical list of stroke centers that had been self-certified/independently designated by the state.
5. For states preliminarily determined not to have a self-certification or independent designation process, we confirmed that there was, in fact, no self-certification or independent designation process. If our preliminary categorization was incorrect and an independent state designation option was available, we followed the process outlined in Step 4.

© 2024 Hsia RY et al. *JAMA Network Open.*

eTable 1. Comparable Stroke Center Certification and Designation Levels Across National Certifying Organizations and States (repurposed from doi:10.1161/STROKEAHA.123.045368)

**Stroke Center Certification/Designation Level**	**Corresponding State(s) and/or National Certifying Organization(s)**
Comprehensive Stroke Center (CSC)<br>This program is the most demanding stroke certification and is designed for those hospitals that have specific abilities to receive and treat the most complex stroke cases.	
Level I	Alabama, Idaho, Mississippi, Missouri, Washington, Texas
Thrombectomy Capable Stroke Center (TSC)<br>This program is designed for hospitals providing endovascular procedures and post-procedural care.	
Level IIa	Alabama
Level II	Missouri, Texas
PSC+	DNV, North Dakota
Primary Stroke Center (PSC)<br>This program is designed for hospitals providing the critical elements to achieve long-term success in improving outcomes for stroke patients.	
Level II	Alabama, Idaho, Mississippi, Washington
Level III	Missouri, Texas
PSC-E	Louisiana (also has PSC)
Acute Stroke Ready Hospital (ASRH)<br>This program is for hospitals or emergency centers with a dedicated stroke-focused program.	
Stroke Ready	ACHC
Level III	Alabama, Idaho, Mississippi, Washington
Level IV	Missouri, Texas
Ar Stroke Ready Hospitals	Arkansas
Remote Treatment Stroke Centers	Georgia
Primary Stroke Service Centers	Massachusetts
Stroke Receiving Facilities	Utah

Note: Comprehensive Stroke Center (CSC), Thrombectomy Capable Stroke Center (TSC), Primary Stroke Center (PSC), and Acute Stroke Ready Hospital (ASRH), are the four levels of advanced stroke center certification recognized by the American Heart Association (AHA), American Stroke Association (ASA), and The Joint Commission (TJC). The definition for each level was obtained from The Joint Commission. Corresponding or similar levels, as identified by state officials, state departments of health, national certifying organizations, and/or state certification or designation criteria, are listed below each Joint Commission-recognized level in the lefthand column. National certifying organizations and states recognizing each of the reported levels are included in the righthand column.

© 2024 Hsia RY et al. *JAMA Network Open.*

eTable 2. Complete Regression Results of the Models Presented in Figures 3 and 4

	**Model 1A**			**Model 1B**		
	Overall	Competing Risk Models		Overall	Competing Risk Models	
	Any level	PSC or higher	ASRH	Any level	PSC or higher	ASRH
Disadvantage status of surrounding neighborhoods						
Mixed communities (reference)	1.00	1.00	1.00	1.00	1.00	1.00
	[1.00,1.00]	[1.00,1.00]	[1.00,1.00]	[1.00,1.00]	[1.00,1.00]	[1.00,1.00]
Most advantaged	1.24**	1.41**	0.48**	1.07	1.06	0.74
	[1.07,1.44]	[1.22,1.62]	[0.29,0.79]	[0.91,1.25]	[0.89,1.25]	[0.43,1.27]
Relatively advantaged	1.25**	1.28**	0.74	1.02	0.95	1.00
	[1.11,1.40]	[1.14,1.43]	[0.52,1.06]	[0.90,1.15]	[0.83,1.08]	[0.69,1.44]
Relatively disadvantaged	0.89*	0.94	0.89	0.80**	0.85**	0.92
	[0.82,0.98]	[0.85,1.03]	[0.74,1.05]	[0.73,0.87]	[0.77,0.93]	[0.77,1.10]
Most disadvantaged	0.43**	0.31**	0.66*	0.58**	0.48**	0.58**
	[0.34,0.55]	[0.21,0.45]	[0.48,0.91]	[0.45,0.74]	[0.34,0.68]	[0.41,0.81]
Located in urban	3.51**	6.61**	0.45**	1.58**	2.31**	0.94
	[3.20,3.84]	[5.84,7.48]	[0.38,0.54]	[1.40,1.77]	[1.93,2.76]	[0.73,1.20]
population size in HSA (log transformed)				1.05**	1.14**	0.90**
				[1.01,1.08]	[1.09,1.19]	[0.85,0.95]
hospital beds (log transformed)				2.10**	2.43**	0.70**
				[2.00,2.20]	[2.24,2.65]	[0.63,0.77]
indicator for missing population				25.42**	55.38**	0.40**
				[19.01,34.00]	[36.17,84.79]	[0.25,0.65]
indicator for missing hospital beds				0.69**	0.86**	0.54**
				[0.62,0.77]	[0.78,0.96]	[0.38,0.75]
N of observations	157,291					
Number of hospitals	5,055					
Number achieving PSC+	2,038					
Number achieving ASRH	602					

Note: Values are coefficient [95% CI]. *p<0.05 ** p<0.01. PSC = Primary Stroke Center. ASRH = Acute Stroke Ready Hospital.

© 2024 Hsia RY et al. *JAMA Network Open.*

eTable 3. Hazard Ratios for Stroke Certification Adoption When Geographic Coverage is Expanded to Include All Residents Within a Hospital Service Area

	**Model 1A**			**Model 1B**		
	Overall	Competing Risk Models		Overall	Competing Risk Models	
	Any level	PSC or higher	ASRH	Any level	PSC or higher	ASRH
Socioeconomic status of hospital neighborhood						
Mixed communities	1.00	1.00	1.00	1.00	1.00	1.00
	[1.00,1.00]	[1.00,1.00]	[1.00,1.00]	[1.00,1.00]	[1.00,1.00]	[1.00,1.00]
Most advantaged	1.69**	1.61**	0.96	1.08	0.98	1.22
	[1.36,2.11]	[1.30,2.00]	[0.54,1.72]	[0.86,1.35]	[0.78,1.24]	[0.66,2.25]
Relatively advantaged	1.21**	1.11	1.18	1.04	0.99	1.34
	[1.05,1.39]	[0.96,1.29]	[0.83,1.68]	[0.90,1.20]	[0.84,1.16]	[0.94,1.93]
Relatively disadvantaged	0.75**	0.79**	0.78**	0.74**	0.83**	0.78**
	[0.68,0.83]	[0.71,0.88]	[0.65,0.93]	[0.68,0.82]	[0.75,0.93]	[0.65,0.94]
Most disadvantaged	0.41**	0.29**	0.59*	0.52**	0.50**	0.51**
	[0.29,0.57]	[0.17,0.51]	[0.38,0.92]	[0.37,0.74]	[0.31,0.82]	[0.33,0.80]
	[0.66,0.83]	[0.82,1.01]	[0.27,0.52]	[0.57,0.71]	[0.72,0.90]	[0.33,0.64]
Urban hospital	3.66**	6.94**	0.43**	1.56**	2.34**	0.90
	[3.35,4.01]	[6.13,7.85]	[0.36,0.51]	[1.39,1.76]	[1.96,2.81]	[0.70,1.15]
population size of hospital service area (log transformed)				1.05**	1.13**	0.89**
				[1.01,1.08]	[1.09,1.18]	[0.84,0.94]
hospital beds (log transformed)				2.11**	2.44**	0.70**
				[2.01,2.21]	[2.24,2.66]	[0.63,0.77]
indicator for missing community disadvantage status	0.74**	0.91	0.38**	0.63**	0.81**	0.46**
indicator for missing population				26.08**	57.97**	0.38**
				[19.54,34.80]	[37.60,89.37]	[0.23,0.62]
indicator for missing hospital beds						
N of observations	157,291					
Number of hospitals	5,055					

Note: Values are coefficient [95% CI]. *p<0.05 ** p<0.01. PSC = Primary Stroke Center. ASRH = Acute Stroke Ready Hospital.

© 2024 Hsia RY et al. *JAMA Network Open.*
