# Supplementary material for: Provision of Stroke Care Services by Community Disadvantage Status in the US, 2009-2022
Source: JAMA Netw Open. 2024 Jul 25;7(7):e2421010. doi: 10.1001/jamanetworkopen.2024.21010 (PMC11273237; doi:10.1001/jamanetworkopen.2024.21010)
Supplement: Supplement 2. — Data Sharing Statement [file jamanetwopen-e2421010-s002.pdf]

Data Sharing Statement

Hsia. Provision of Stroke Care Services by Community Disadvantage Status in the US, 2009-2022. *JAMA Netw Open*. Published July 25, 2024. doi:10.1001/jamanetworkopen.2024.21010

Data
Data available: No
